# The Impacts of Sortase A and the 4′-Phosphopantetheinyl Transferase Homolog Sfp on *Streptococcus mutans* Extracellular Membrane Vesicle Biogenesis

**DOI:** 10.3389/fmicb.2020.570219

**Published:** 2020-10-26

**Authors:** Joyce C. Morales-Aparicio, Patricia Lara Vasquez, Surabhi Mishra, Ana L. Barrán-Berdón, Manasi Kamat, Kari B. Basso, Zezhang T. Wen, L. Jeannine Brady

**Affiliations:** ^1^Department of Oral Biology, University of Florida, Gainesville, FL, United States; ^2^Department of Chemistry, University of Florida, Gainesville, FL, United States; ^3^Department of Oral and Craniofacial Biology, Louisiana State University Health Sciences Center New Orleans, New Orleans, LA, United States; ^4^Department of Microbiology, Immunology, and Parasitology, Louisiana State University Health Sciences Center New Orleans, New Orleans, LA, United States

**Keywords:** vesicles, membrane, proteome, lipidome, *Streptococcus mutans*

## Abstract

Extracellular membrane vesicles (EMVs) are produced by many Gram-positive organisms, but information regarding vesiculogenesis is incomplete. We used single gene deletions to evaluate the impacts on *Streptococcus mutans* EMV biogenesis of Sortase A (SrtA), which affects *S. mutans* EMV composition, and Sfp, a 4′-phosphopantetheinyl transferase that affects *Bacillus subtilis* EMV stability. Δ*srtA* EMVs were notably larger than Δ*sfp* and wild-type (WT) EMVs. EMV proteins identified from all three strains are known to be involved in cell wall biogenesis and cell architecture, bacterial adhesion, biofilm cell density and matrix development, and microbial competition. Notably, the AtlA autolysin was not processed to its mature active form in the Δ*srtA* mutant. Proteomic and lipidomic analyses of all three strains revealed multiple dissimilarities between vesicular and corresponding cytoplasmic membranes (CMs). A higher proportion of EMV proteins are predicted substrates of the general secretion pathway (GSP). Accordingly, the GSP component SecA was identified as a prominent EMV-associated protein. In contrast, CMs contained more multi-pass transmembrane (TM) protein substrates of co-translational transport machineries than EMVs. EMVs from the WT, but not the mutant strains, were enriched in cardiolipin compared to CMs, and all EMVs were over-represented in polyketide flavonoids. EMVs and CMs were rich in long-chain saturated, monounsaturated, and polyunsaturated fatty acids, except for Δ*sfp* EMVs that contained exclusively polyunsaturated fatty acids. Lipoproteins were less prevalent in EMVs of all three strains compared to their CMs. This study provides insight into biophysical characteristics of *S. mutans* EMVs and indicates discrete partitioning of protein and lipid components between EMVs and corresponding CMs of WT, Δ*srtA*, and Δ*sfp* strains.

## Introduction

Extracellular membrane vesicles (EMVs) are non-replicative membrane-bound nanostructures used as a secretion system in bacteria (Bitto and Kaparakis-Liaskos, 2017). Bacterial EMVs were first studied exclusively in Gram-negative bacteria, presuming that the lack of an outer membrane and presence of a thick cell wall would preclude Gram-positive bacteria from releasing them. However, EMV production has now been demonstrated for Gram-positive bacteria as well as fungi that also have a thick cell wall (reviewed in Brown et al., 2015). Although the mechanisms of secretion are not yet defined, several have been proposed, including degradation of peptidoglycan, membrane blebbing, cell death, turgor pressure, and use of membrane-localized protein channels (Brown et al., 2015; Toyofuku et al., 2018). EMVs have been hypothesized to contribute to horizontal gene transfer, phage susceptibility, microbial survival, competition, pathogenicity, colonization, and biofilm development (Biagini et al., 2015; Grande et al., 2017; Liu et al., 2018; Toyofuku et al., 2018; Nagakubo et al., 2020).

Tooth decay is the most common infectious disease in the world, resulting in enormous associated health care costs (Klein et al., 2015; Marcenes et al., 2013). *Streptococcus mutans* is a major cariogenic Gram-positive bacterium and member of the oral microbiota. Among its pathogenic factors are its abilities to adhere to and colonize the tooth surface, to form tenacious biofilms, to ferment various dietary sugars to produce acidic end products, to tolerate a low pH environment, to produce bacteriocins (mutacins) to outcompete other oral bacteria, and to exchange genetic material via natural genetic competence (Hamada and Slade, 1980; Carlsson, 1994; Li et al., 2001; Krzysciak et al., 2014; Liao et al., 2014). *S. mutans* adhesion involves both sucrose-dependent and sucrose-independent mechanisms (Krzysciak et al., 2014). Glucosyltransferases (Gtfs) and glucan-binding proteins (Gbps) are major contributors to sucrose-dependent adhesion that form glucans from sucrose and attach these products to the bacterial surface, respectively (Krzysciak et al., 2014). Other cell wall-linked proteins, including surface protein P1 (also called SpaP, Antigen I/II, PAc) and Wall-Associated Protein A (WapA), serve as adhesins that promote *S. mutans* binding to salivary constituents in the acquired pellicle in the absence of sucrose (Krzysciak et al., 2014; Liao et al., 2014). Sortase A (SrtA) is a membrane-localized transpeptidase that cleaves its substrate proteins, including P1, WapA, WapE, GbpC, DexA, and FruA (Ajdić et al., 2002), at a consensus motif near the carboxy-terminus, and subsequently anchors the secreted extracellular polypeptide via covalent linkage to the cell wall peptidoglycan (Cossart and Jonquières, 2000). Deletion of *srtA* results in release of normally cell wall-anchored proteins into the extracellular environment, and causes a pronounced defect in *S. mutans* biofilm formation (Lee and Boran, 2003; Lévesque et al., 2005). Although SrtA deficiency does not prevent EMV production, deletion of *srtA* was shown previously to result in differences in the protein composition of *S. mutans* EMVs and significantly reduced extracellular DNA (eDNA) in planktonic and biofilm cultures (Liao et al., 2014), hence inclusion of this mutant in the current study.

*Streptococcus mutans* EMVs were first reported in 2014 and shown to carry eDNA as well as proteins that aid in biofilm formation (Liao et al., 2014). *Streptococcus pyogenes*, *Streptococcus pneumoniae*, and *Staphylococcus aureus* also produce EMVs (Lee et al., 2009; Olaya-Abril et al., 2014; Biagini et al., 2015; Resch et al., 2016). The first Gram-positive organism proven to produce EMVs, *Bacillus anthracis*, carries bacterial toxins within the vesicles, and immunization of mice with EMVs confers protection against bacterial challenge (Rivera et al., 2010). In studies to characterize the nature of bacillus vesicles, it was found that surfactin represented an important mechanistic component. Surfactin, also known as subtilysin, is an amphiphilic antimicrobial cyclic lipopeptide and powerful biosurfactant that inhibits fibrin clot formation and lyses bacterial protoplasts and spheroplasts by inducing cationic channels in lipid bilayer membranes (Bernheimer and Avigad, 1970; Maget-Dana and Ptak, 1995; Yuan et al., 2017; Bucci et al., 2018). In *Bacillus subtilis*, *sfp* encodes a 4′-phosphopantetheinyl transferase that transfers a phosphopantetheine group from coenzyme A to a peptidyl carrier protein during biosynthesis of the surfactin lipopeptide, as well as that of the siderophore, bacillibactin (Nakano et al., 1988; Brown et al., 2014). *B. subtilis*Δ*sfp* strains display a defect in biofilm formation despite hyperaccumulation of EMVs because surfactin contributes to EMV rupture and cargo release in this organism (Brown et al., 2014). In *S. mutans*, the *sfp* homolog *mubP* (*Smu_*1334c) is located within a prevalent large genomic island called TnSmu2. This locus harbors a gene cluster that encodes non-ribosomal peptide synthetases (NRPSs), polyketide synthases (PKSs), and accessory and regulatory factors also involved in NRP and PK biosynthesis (Wu et al., 2010). Although the sequences of these genes and their locations and organization differ among *S. mutans* strains, each TnSmu2 genomic island encodes NRPS, PKS, and accessory proteins. The Mub designation stems from the hybrid NRP/PK pigment referred to as mutanobactin that is reported to contribute to oxygen and H_2_O_2_ tolerance (Wu et al., 2010). Also encoded within the TnSmu2 genomic island are acyl carrier proteins, bacitracin and gramicidin synthase homologs, thioesterases, ABC transporters, transcriptional regulators, and two-component response regulators.

To better understand vesiculogenesis and the function of *S. mutans* EMVs, the goals of the current study were to isolate and characterize the physiochemical properties of EMVs produced by wild-type (WT), Δ*srtA*, and Δ*sfp* strains, and to compare the proteomes and lipidomes of EMVs derived from the three strains with one another and with those of the corresponding cytoplasmic membranes (CMs).

## Materials and Methods

### Bacterial Strains and Cultivation

*Streptococcus mutans* serotype c WT strain NG8 (Knox et al., 1986), its Δ*srtA* mutant, strain PC339 (Oli et al., 2012), and its Δ*sfp* mutant were used in this study. NG8 and UA159 are both widely used lab strains whose genomes have both been sequenced^1^. To ensure consistency in parental background, a new NG8-based Δ*sfp* strain was generated for this study from an allelic exchange mutant originally made in strain UA159 in which the *sfp*-coding sequence was replaced with a non-polar kanamycin resistance element (npk) (Zeng et al., 2006; Wen et al., in press). The ∼1 kilobase chimeric Δ*sfp*:npk fragment including 5’ and 3’ flanking regions was amplified using high fidelity DNA polymerase Q5 (New England Biolabs) and primers 5′-acacagagactctacgacaagc and 3′-acacttgcaaattcagtcagagac, verified by Sanger sequencing, and following ligation with a npk, used to transform strain NG8 with inclusion of competence stimulating peptide (Li et al., 2001). The resulting Δ*sfp* mutant was selected on BHI agar plates containing kanamycin (1 mg/mL). Because the flanking genes are transcribed in the same orientation as *sfp*, and the 236 bp downstream gene sequence includes its own promoter, replacement of the *sfp-*coding sequence with a commonly used non-polar element will have little or no effect on the transcription of adjacent genes. All strains were maintained in BHI medium with kanamycin (1 mg/mL) and/or erythromycin (Erm, 10 mg/mL), as appropriate. Bacteria were cultured in the chemically defined medium of Terlejcky (TDM) for EMV and CM preparations (Terleckyj et al., 1975).

### Isolation of EMVs and Preparation of CMs

Overnight cultures of *S. mutans* strains were grown at 37°C in 40 mL Todd–Hewitt broth with 3% yeast extract in a 5% CO_2_ chamber, then 10 mL was transferred to 1 L of TDM and grown at 37°C for 16 h. For EMV isolation, bacterial cells were pelleted by centrifugation at 18,000 × *g* for 30 min at 4°C. Supernatants were filtered through a 0.2 μM polyethersulfone membrane (Thermo Scientific^TM^ Nalgene^TM^ Rapid-Flow^TM^), and concentrated ∼50-fold using a Stirred Ultrafiltration Cell and 100 kDa cutoff membrane (Ultrafiltration Discs, PLHK, Ultracel regenerated cellulose, Millipore). Concentrated supernatants were centrifuged at 100,000 × *g* for 1 h at 4°C, and crude vesicle pellets were resuspended in 1 mL of 35% OptiPrep^TM^ (Sigma) in phosphate buffered saline (PBS) and overlaid on a 10–30% OptiPrep step gradient with 5% increments in 5 mL 13 × 51 mm open top thin-walled tubes (Nalgene), and centrifuged at 140,000 × *g* for 16 h at 4°C. Five 1 mL fractions were removed from the top of each tube. Vesicle-containing fractions were identified by SDS-PAGE and transmission electron microscopy (TEM) as described previously (Liao et al., 2014) (fractions 4 and 5 for each strain), pooled, diluted to ∼20 mL in PBS, then centrifuged at 100,000 × *g* for 2 h at 4°C to separate vesicles from the OptiPrep^TM^, and EMVs resuspended in 0.5 mL of PBS. Bacterial cells harvested from the same cultures were used to prepare CM as previously described (Mishra et al., 2019).

### SDS-PAGE and Western Blot Analysis

SDS-PAGE and Western blotting were performed as previously described (Liao et al., 2014) using a 1:500 dilution of polyclonal rabbit antisera against purified recombinant Smu_63c, AtlA, GbpB, YidC1, YidC2, Ffh, FtsY, and SecA. Horseradish-peroxidase conjugated goat anti-rabbit IgG secondary antibody (MP Biomedicals) was used at a 1:1000 dilution. Antibody reactivity was traced using a chemiluminiscent detection kit (Amersham^TM^ ECL^TM^ Prime Western Blotting Detection Reagent, GE Healthcare).

### Nanoparticle Tracking Analysis (NTA)

Extracellular membrane vesicle particle size was analyzed by nanoparticle tracking analysis (NTA) using NS300 (Malvern Panalytical, Malvern, United Kingdom). Samples were diluted 100–1000-fold in PBS, and ∼1 mL of diluted sample was manually injected into the instrument. Five videos of 60 s each were recorded. Size distribution and particle concentration were analyzed using NanoSight NTA (v3.4) software.

### Dynamic Light Scattering (DLS) and ζ-Potential

Dynamic light scattering (DLS) and ζ-potential measurements were performed using ZetaPALS (Brookhaven Instruments). Samples were diluted threefold in 20 mM HEPES to reach the required measurement volume, and evaluated at 25°C. For ζ-potential, an Aqueous Zeta Potential Electrode (BI-ZEL, Brookhaven Instruments) was used in 10 mm, 4.5 mL clear polystyrene cuvettes (BI-SCP, Brookhaven Instruments). Measurements of 10 runs of 30 cycles each at 25°C were taken.

### Lipid and Protein Extractions

A Bligh and Dyer method (Classics Bligh and Dyer, 1959) was used in which proteins localize to the middle water layer, lipids to the bottom chloroform layer, and metabolites to the top methanol layer. Briefly, 2 mL of cold chloroform/methanol (2:1) was added to 150 μL of each EMV or CM sample and vortexed for one minute. Next 500 μL of water was added, chilled at 4°C, then vortexed again. Samples were centrifuged for 10 min at 1300 × *g*, and the white protein “disc” visible in the water layer was carefully transferred to an Eppendorf tube and dried in a speed vac. The bottom chloroform layer was transferred into a new clean HPLC vial.

### Impact II QTOF LC-MS/MS for Analysis of Lipids

Capillary-liquid chromatography tandem mass spectrometry (Cap-LC-MS) for analysis of lipids was performed on a Bruker Daltonics, Impact II quadrupole time-of-flight (QTOF) mass spectrometer equipped with an Apollo II ion funnel ESI source (Bruker) operated in positive ion mode. The LC system was an UltiMate^TM^ 3000 RSLCnano system from Thermo Scientific. The mobile phase A consisted of 50% acetonitrile in water with 10 mM ammonium formate and 0.1% formic acid; the mobile phase B was 90% isopropyl alcohol in acetonitrile with 10 mM ammonium formate and 0.1% formic acid. A 5 μL volume of sample was loaded on to the m-Precolumn [Thermo Scientific, C18 PepMap 100, (5 μm, 100 Å)] and washed with mobile phase A at a flow rate of 25 μL/min. This was held for 10 min and washed with 2% B to desalt and concentrate the sample. The injector port was switched to inject, and the sample was eluted off of the trap onto the column. A ThermoScientific Acclaim PepMap RSLC, C18 UHPLC, 300 μm × 15 cm, 2 μm, 100 Å was used for chromatographic separation. Lipids were eluted directly off the column into the Q-TOF system using a gradient of 0–5 min: 50% B, 5–50 min: 98% B, 50–70 min: 98% B, 70–75 min: 98–50% B, 75–90 min: 50% B at 5 μL/min flow rate. The column was maintained at 40°C. The electrospray source was operated at with a spray voltage of 4.5 kV, a capillary gas temperature of 200°C, drying gas (N_2_) at 4.0 L/min, nebulizer at 0.3 bar. The analysis was programmed for a full scan recorded between mass range *m/z* 350–3000. The full spectral rate was 2 Hz.

### Protein Digestion

Total protein was determined using a Qubit 3 Fluorometer (Invitrogen by Thermo Fisher Scientific) and the appropriate volume of each sample was taken to equal 10 μg total protein for digestion. Samples were digested with sequencing grade trypsin/lys C rapid digestion kit from Promega (Madison, WI, United States) per manufacturer’s protocol. Rapid digestion buffer (provided with the kit) was added to the sample three times the sample volume, and each sample was reduced with dithiothreitol (DTT) (0.1 M in 100 mM ammonium bicarbonate) for 30 min at 56°C and then alkylated with Iodoacetamide (55 mM in 100 mM ammonium bicarbonate) for 30 min in dark at room temperature. The enzyme, trypsin/lys C was then added to the samples in 1:10 (enzyme to substrate) ratio and incubated at 70°C for 1 h. The digestion was terminated by addition of trifluoroacetic acid (TFA). The MS analysis is immediately performed to ensure high-quality tryptic peptides with minimal non-specific cleavage.

### Q Exactive HF Orbitrap LC-MS/MS for Analysis of Proteins

Nano-liquid chromatography tandem mass spectrometry (Nano-LC/MS/MS) was performed using a Thermo Scientific Q Exactive HF Orbitrap mass spectrometer equipped with an EASY Spray nanospray source (Thermo Scientific) operated in positive ion mode. The LC system was an UltiMate^TM^ 3000 RSLCnano system from Thermo Scientific. The mobile phase A was water containing 0.1% formic acid and the mobile phase B was acetonitrile with 0.1% formic acid. Injection volume was calculated for each sample such that that 0.2 μg of sample was injected on to a Thermo Scientific Acclaim Trap Cartridge (C18 column, 75 μm ID, 2 cm length, 3 mm 100 Å pore size) at a 5 μL/min flow rate. This was held for 10 min and washed with 2% mobile phase B to desalt and concentrate peptides. The injector port was programmed to switch to inject, and peptides were eluted off the trap onto the nanocolumn (Thermo Scientific, C18, 75 mm ID, 25 cm length, 3 mm, 100 Å pore size) at a flow rate of 300 nL/min using the following gradient—0–10 min: 2% B, 10–20 min: 2–7% B, 20–240 min: 7–45% B, 240–250 min: 45–80% B, 250–255 min: 80% B, 255–256 min: 80–2% B, 256–260 min: 2% B. Column temperature was maintained 35°C. Total run time was 260 min. The MS/MS was acquired according to standard conditions established in the lab. The EASY Spray source operated with a spray voltage of 1.5 kV and a capillary temperature of 200°C. The scan sequence of the mass spectrometer was based on the original TopTen^TM^ method (Kelstrup et al., 2018). The analysis was programmed for a full scan recorded between 375 and 1575 Da at 60,000 resolution, and a MS/MS scan at resolution 15,000 to generate product ion spectra to determine amino acid sequence in consecutive instrument scans of the fifteen most abundant peaks in the spectrum. The AGC Target ion number was set at 3e6 ions for full scan and 2e5 ions for MS^2^ mode. Maximum ion injection time was set at 50 ms for full scan and 55 ms for MS^2^ mode. Micro scan number was set at 1 for both full scan and MS^2^ scan. The HCD fragmentation energy (N)CE/stepped NCE was set to 28. Singly charged ions were excluded from MS^2^. Dynamic exclusion was enabled with a repeat count of 1 within 30 s and to exclude isotopes. A Siloxane background peak at 445.12003 was used as the internal lock mass.

HeLa protein digest standard was used to evaluate integrity and performance of the columns and mass spectrometer. If the number of protein ID’s from the HeLa standard fell below 2700, the instrument was cleaned and new columns installed.

### Database Searching

For the metabolomic statistical analyses, data were converted to mzxml format using MS Convert (proteowizard open source program) (Kessner et al., 2008; Chambers et al., 2012). The mzxml files were uploaded to XCMS (Scripps Research Institute) (Tautenhahn et al., 2012; Patti et al., 2013; Gowda et al., 2014) and a Multi-way job was submitted using the preprogrammed HPLC/QTOF parameters. The results were shared and can be accessed anytime. For proteomics, all MS/MS data were analyzed using Proteome Discoverer software (Thermo Fisher Scientific, San Jose, CA, United States; version IseNode in Proteome Discoverer 2.2.0.388) Sequest HT was used as the database search algorithm. Sequest (XCorr Only) was set up to search *S. mutans* NG8 assuming the digestion enzyme trypsin. Sequest (XCorr Only) was searched with a fragment ion mass tolerance of 0.020 Da and a precursor ion tolerance of 10.0 ppm. Carbamidomethyl of cysteine was specified in Sequest (XCorr Only) as a fixed modification. Deamidation of asparagine and oxidation of methionine were specified in Sequest (XCorr Only) as variable modifications.

### Proteome and Lipidome Analyses

Scaffold (version Scaffold_4.9.0, Proteome Software Inc., Portland, OR, United States) was used to validate MS/MS based peptide and protein identifications. Peptide identifications were accepted if they could be established at greater than 95.0% probability by the Peptide Prophet algorithm (Keller et al., 2002) with Scaffold delta-mass correction. Protein identifications were accepted if they could be established at greater than 99.0% probability and contained at least one identified peptide. Protein probabilities were assigned by the Protein Prophet algorithm (Nesvizhskii et al., 2003). Proteins that contained similar peptides and could not be differentiated based on MS/MS analysis alone were grouped to satisfy the principles of parsimony. Proteins sharing significant peptide evidence were grouped into clusters. Lipid data were analyzed using the MetaboAnalyst 4.0 web-based software from McGill University using FDR adjusted *p*-value^2^ (Chong et al., 2019). Lipids with an FDR adjusted *p*-value ≤ 0.1 and fold-change ≥ 1.5 were considered significant for further consideration of over-represented lipids in EMVs compared to CMs.

### Bioinformatic Analyses

The transmembrane (TM) domain prediction web tool (TMHMM; Krogh et al., 2001) was used to identify and characterize predicted integral membrane proteins. Proteome functional analysis was made using the Database for Annotation, Visualization, and Integrated Discovery (DAVID) (Huang Da et al., 2009). SignalP version 4.0 was used for signal peptide prediction (Petersen et al., 2011). Venn diagrams were created using the Meta-Chart web tool (Meta-Chart, 2020). Proteomic data were submitted to PRIDE (Côté et al., 2012) data repository via ProteomeXchange with identifier PXD019825. Lipidomic data were submitted to MetaboLights (Kale et al., 2016) data repository with identifier MTBLS1812.

## Results

### Isolation of *S. mutans* Extracellular Membrane Vesicles

Extracellular membrane vesicles were isolated by ultracentrifugation and density gradient from the filtered supernatants of quadruplicate stationery-phase planktonic cultures of *S. mutans* WT, and Δ*srtA* and Δ*sfp* mutant strains grown in a defined medium previously developed for streptococci (Terleckyj et al., 1975). In addition, protoplasts derived from bacterial cells harvested from the same cultures were used to prepare corresponding CMs (Mishra et al., 2019). Similar to the vesicle pellets described for *S. pneumoniae* (Olaya-Abril et al., 2014), and the *B. subtilis*Δ*sfp* mutant but not WT strain (Brown et al., 2014), the pelleted material containing *S. mutans* EMVs was brown in color. The average bacterial wet weight pellet yields were 2.33 ± 0.04, 2.43 ± 0.04, and 2.33 ± 0.03 g, and EMV wet weight pellet yields were 147.6 ± 30, 162.9 ± 65.5, and 114.45 ± 8.55 mg for WT, Δ*srtA*, and Δ*sfp* strains, respectively. Following isolation by Optiprep^TM^ density gradient, EMV-containing fractions were identified by SDS-PAGE and TEM analyses (data not shown), with EMVs consistently localized to fractions 4 and 5. These fractions were pooled for further characterization.

### Characterization of Physical Properties of EMVs

**FIGURE 1:**
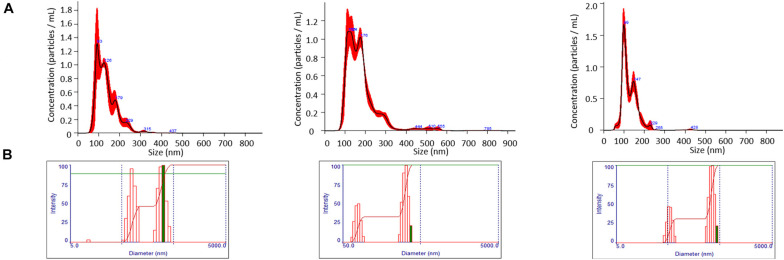
Evaluation of size and particle yield of EMVs. **(A)** Nanoparticle tracking analysis demonstrates particle yields of 1.15 × 10^11^ ± 1.09 × 10^10^, 1.46 × 10^11^ ± 1.10 × 10^8^, and 5.94 × 10^10^ ± 5.98 × 10^9^ particles/mL with mean EMV diameters of 131.8 ± 5.8, 174.5 ± 3.85, 131.45 ± 6.3 nm for WT, Δ*srtA*, and Δ*sfp* strains, respectively. **(B)** Dynamic light scatter (DLS) analysis demonstrates a bimodal distribution of EMVs for all three strains. DLS signal intensity is reflective of particle size, not particle number.

When evaluated by NTA, average EMV diameters of 131.8 ± 5.8, 174.5 ± 3.85, and 131.45 ± 6.3 nm were measured for the WT, Δ*srtA*, and Δ*sfp* strains, respectively (Figure 1A). When evaluated by uranyl acetate negative staining and TEM, EMV size estimates of 20–30, 50–60, and 30–40 nm for the WT, Δ*srtA*, and Δ*sfp* strains, respectively, were considerably smaller. Desiccation during TEM sample preparation likely accounts for this pronounced size difference (Brown et al., 2014). NTA also provided information regarding EMV quantity detected in each sample: 1.46 × 10^11^ ± 1.10 × 10^8^ particles/mL for the Δ*srtA* mutant, 1.15 × 10^11^ ± 1.09 × 10^10^ for the WT strain, followed by 5.94 × 10^10^ ± 5.98 × 10^9^ for the Δ*sfp* mutant. EMVs were further characterized by DLS. Intensity of DLS signals relates to particle size rather than to particle number. When evaluated by this method, a bimodal distribution was observed for each strain’s EMVs with discrete populations at ∼50 and ∼500 nm (Figure 1B). Again, the largest mean EMV diameter, 168.3 ± 1.6 nm, was observed for the Δ*srtA* mutant, followed by the Δ*sfp* mutant strain at 159.3 ± 16 nm, and the WT strain at 129.9 ± 7.0 nm. DLS polydispersity indices were 0.273, 0.283, and 0.346 for WT, Δ*srtA*, and Δ*sfp* EMVs, respectively. These values suggest heterogeneity within the samples, as an index of <0.1 is considered to represent a monodisperse and homogeneous preparation (Clayton et al., 2016). The higher polydispersity index measured for the Δ*sfp* strain may explain differences in size measured by NTA compared to by DLS.

**FIGURE 2:**
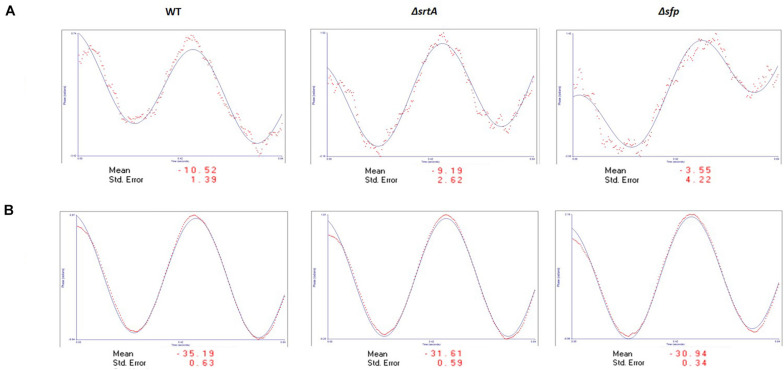
ζ-Potential analysis of *S. mutans* EMVs. **(A)** EMVs demonstrate a slight negative charge approaching electroneutrality. **(B)** Protoplasts from all three strains demonstrate a negative electrical charge of approximately –30 to –35 mV.

ζ-Potential measurements reflect electrostatic repulsion and provide information regarding stability of a colloidal suspension. Generally, the larger the ζ-potential value, the more stable the sample (Greenwood and Kendall, 1999). The closer the ζ-potential value is to zero, the more prone the sample is to agglomeration (Du Plessis et al., 1996). ζ-Potential measurements were performed on EMV samples, as well as on protoplasts derived from the corresponding bacterial cells (Figure 2). The figure shows the sinusoidal distribution of the electrophoretic mobility of the applied electric field. Henry’s equation was used to determine zeta potential values (Kaszuba et al., 2010). The EMV ζ-potential values were −10.52, −9.19, and −3.55 mV for the WT, Δ*srtA*, and Δ*sfp* EMVs, respectively. Thus, the EMVs of all three strains approached electroneutrality with the Δ*sfp* mutant value closest to zero. In contrast, protoplasts of all three strains had pronounced negative charges: −35.19, −31.61, and −30.94 mV for WT, Δ*srtA*, and Δ*sfp* strains, respectively. These results indicate that *S. mutans* protoplasts are more stable in suspension than corresponding EMVs derived from the same bacterial cultures.

### Proteomic Comparison of *S. mutans* EMVs and CM Preparations

**FIGURE 3:**
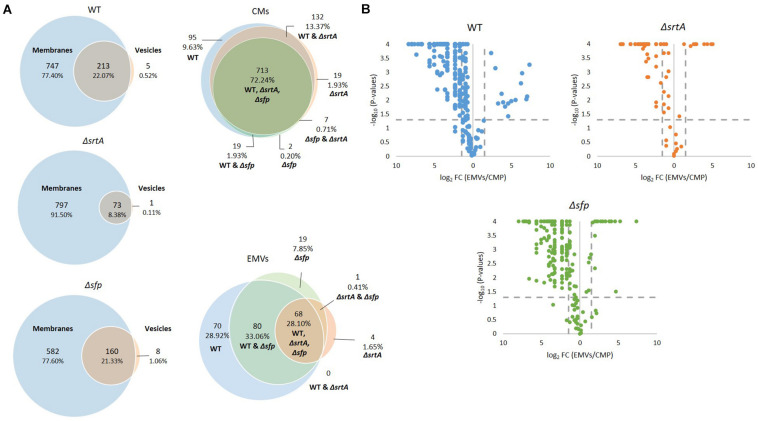
Comparison of protein content of EMVs with corresponding cytoplasmic membranes. **(A)** Venn diagram comparing proteins detected in cytoplasmic membranes (CMs) and corresponding EMVs, and in all three strains’ EMVs and CMs. **(B)** Volcano plots of *p*-values vs. fold-change to compare protein profiles of CMs (left of *Y*-axis) with corresponding EMVs (right of *Y*-axis) from each strain.

In composite, a total of 987 proteins were collectively identified by mass spectrometry in all CM samples, and 242 proteins in all EMV samples (Supplementary Table S1). The degree of overlap in protein composition of EMVs compared to corresponding CMs for each strain, as well as three-way comparisons of EMVs and CMs from all three strains, are illustrated by Venn diagram (Figure 3A). A given protein was considered as present when it was detected in three of four of the individual replicates. A total of 960, 870, and 742 individual proteins were identified in CMs from the WT, Δ*srtA*, and Δ*sfp* strains, respectively, with far more proteins unique to CMs compared to EMVs for each strain. A strikingly smaller subset of proteins was shared between Δ*srtA* EMVs and CMs (8.38%) compared to the WT (22.07%) and Δ*sfp* strains (21.33%). This indicates that as previously reported (Liao et al., 2014), the absence of SrtA has a pronounced impact on the protein content of *S. mutans* EMVs. While 72.24% of CM proteins overlapped among the three strains, only 28.10% of EMV proteins were common to all of them. That is, a greater degree of strain-to-strain variability was observed in EMV protein content compared to CM protein content. WT and Δ*sfp* EMV proteins were more similar to one another (61.16%) than were those of WT compared to Δ*srtA* (28.10%), or Δs*rtA* compared to Δ*sfp* (28.51%). In contrast, WT and Δ*sfp* CM proteins were 74.17% similar; WT compared to Δ*srtA* were 85.61% similar; and Δ*srtA* compared to Δ*sfp* were 72.95% similar. Taken together, these results illustrate that the delivery of proteins to *S. mutans* EMVs is a selective process, which does not simply mirror CMs, and is substantially influenced by the presence of SrtA and to a lesser extent Sfp.

In addition to presence and absence, differences in the relative abundance of individual proteins identified in both EMV and CM samples were calculated and displayed graphically as volcano plots of fold-change vs. *p*-value (Figure 3B). Proteins with no change in EMV compared to CM abundance were excluded from these plots. This analysis further demonstrates that deficiency of *srtA* skewed protein content of *S. mutans* EMVs more so than did deficiency of *sfp*, and that this was not a simple consequence of overall changes in CM protein content. To gain insight into the role *S. mutans* EMVs play in delivering specific protein cargo to the extracellular environment, proteins exclusive to the EMVs or with a fold-change of log2 > 1.5 in EMVs compared to CMs, were tabulated (Table 1). These results illustrate that *S. mutans* MV cargo contributes to cell wall development and cell architecture (Wen et al., 2018), bacterial adhesion (Matsumoto-Nakano, 2018), biofilm cell density (Besingi et al., 2017) and matrix development (Bowen and Koo, 2011), and intermolecular competition with other microorganisms (Hamada and Ooshima, 1975). Among the over-represented proteins in the Δ*sfp* EMVs were BacA (Smu_1340) and BacA2 (Smu_1342) that similar to Sfp are also encoded by genes within the TnSmu2 genomic island. BacA and BacA2 were detected in WT and Δ*srtA* CMs, but not in WT EMVs, and were present but not over-represented in Δ*srtA* EMVs.

**TABLE 1:** Proteins over-represented in *S. mutans* EMVs compared to corresponding cytoplasmic membranes.

Gene name	Identified proteins	Accession number	Molecular weight	*p*-Value	Fold change
**Unique and over-represented proteins in WT vesicles**					
Smu_1904c	Bacteriocin	AMF86525.1	37 kDa	0.0001	Vesicles only
PotD	Spermidine/putrescine ABC transporter substrate-binding protein	AMF85492.1	41 kDa	0.21	Vesicles only
Smu_367	Hydrolase	AMF86015.1	22 kDa	0.0001	Vesicles only
FruA	Glycosyl hydrolase family 32	AMF86222.1	159 kDa	0.059	Vesicles only
GbpD	Glucan-binding protein	AMF85671.1	80 kDa	0.0075	Vesicles only
Ftf/Smu_2028	Levansucrase	AMF86409.1	87 kDa	0.00054	160
gbpC	Glucan-binding protein	AMF86686.1	63 kDa	0.0091	120
SagA/GbpB	Peptidoglycan hydrolase	AMF86264.1	45 kDa	0.0011	83
AtlA	Autolysin	AMF85735.1	107 kDa	0.0025	75
Smu82_1213c	Bifunctional metallophosphatase/5′-nucleotidase	AMF85297.1	79 kDa	0.01	46
GtfC	Glucosyltransferase	AMF85466.1	163 kDa	0.011	31
SpaP	Cell wall-linked adhesin	AMF85804.1	170 kDa	0.037	24
GtfB	Glucosyltransferase	AMF85467.1	166 kDa	0.017	18
Smu_63c	Signal peptide protein	AMF86234.1	64 kDa	0.012	15
DexA	Dextranase	AMF86396.1	100 kDa	0.006	13
GtfD	Glucosyltransferase	AMF85549.1	163 kDa	0.013	9.6
Smu_1733c	RNA helicase	AMF86636.1	119 kDa	0.00072	7.5
Smu_609	Cell wall protein precursor	AMF85805.1	79 kDa	0.00021	5.1
**Unique and over-represented proteins in Δ*srtA* vesicles**					
Smu_1904c	Bacteriocin	AMF86525.1	37 kDa	0.0015	Vesicles only
GbpC	Glucan-binding protein	AMF86686.1	63 kDa	0.0001	33
Ftf/Smu_2028	Levansucrase	AMF86409.1	87 kDa	0.0001	28
AtlA	Autolysin	AMF85735.1	107 kDa	0.0001	20
SagA/GbpB	Peptidoglycan hydrolase	AMF86264.1	45 kDa	0.0001	15
GtfC	Glucosyltransferase	AMF85466.1	163 kDa	0.0001	7.5
DexA	Dextranase	AMF86396.1	100 kDa	0.0001	7.3
Smu82_1213c	Bifunctional metallophosphatase/5′-nucleotidase	AMF85297.1	79 kDa	0.0001	6.7
GtfB	Glucosyltransferase	AMF85467.1	166 kDa	0.00012	4.4
**Unique and over-represented proteins in Δ*sfp* vesicles**					
Smu_963c	Deacetylase	AMF85504.1	33 kDa	0.038	Vesicles only
Smu_1904c	Bacteriocin	AMF86525.1	37 kDa	0.0001	Vesicles only
Smu_172	Cell division protein FtsW	AMF86157.1	9 kDa	0.031	Vesicles only
Smu_367	Hydrolase	AMF86015.1	22 kDa	0.0001	Vesicles only
BacA2	Non-ribosomal peptide synthetase	AMF86652.1	186 kDa	0.19	Vesicles only
BacA	Non-ribosomal peptide synthetase	AMF85178.1	314 kDa	0.15	Vesicles only
WapE	Cell wall protein	AMF85397.1	55 kDa	0.0047	Vesicles only
FruA	Glycosyl hydrolase	AMF86222.1	159 kDa	0.26	Vesicles only
GbpD	Glucan-binding protein	AMF85671.1	80 kDa	0.0001	37
SagA/GbpB	Peptidoglycan hydrolase	AMF86264.1	45 kDa	0.0001	24
AtlA	Autolysin	AMF85735.1	107 kDa	0.0001	17
gbpC	Glucan-binding protein	AMF86686.1	63 kDa	0.0001	17
Ftf/Smu_2028	Levansucrase	AMF86409.1	87 kDa	0.0001	14
GtfD	Glucosyltransferase	AMF85549.1	163 kDa	0.0001	7.8
Smu_63c	Signal peptide protein	AMF86234.1	64 kDa	0.0001	6.6
GtfC	Glucosyltransferase	AMF85466.1	163 kDa	0.0001	5
Smu82_1213c	Bifunctional metallophosphatase/5′-nucleotidase	AMF85297.1	79 kDa	0.00032	3.9
GtfB	Glucosyltransferase	AMF85467.1	166 kDa	0.0001	3.9
BrpA	Surface-associated protein	AMF85980.1	44 kDa	0.00011	3.1

*Underlined proteins are present in EMVs of all three strains.*

### Western Blot Analysis of EMVs

**FIGURE 4:**
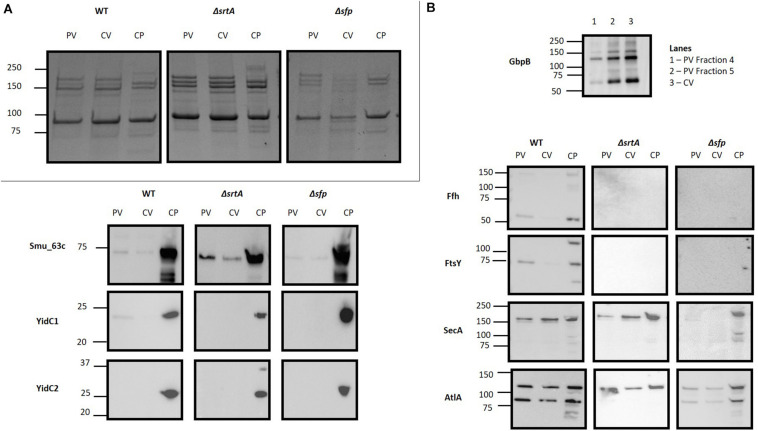
Evaluation of *S. mutans* membrane vesicle by SDS-PAGE and Western blot. **(A)** SDS-PAGE of *S. mutans* bacterial cell pellet (CP), crude vesicle prep (CV) and purified EMVs (PV) of WT, Δ*srtA* and Δ*sfp* mutant strains by SDS-PAGE. **(B)** Corresponding Western blots of the samples shown in **(A)**. Blots were reacted with polyclonal rabbit antibodies raised against the indicated proteins. Due to limited antibody availability, only crude vesicles and Optiprep^TM^ gradient fractions of the WT EMVs were evaluated for GbpB.

Prior to evaluation by mass spectrometry, EMVs were initially evaluated by SDS-PAGE (Figure 4A) and Western blot analysis using a battery of available antibodies against both membrane-localized and extracellular proteins (Figure 4B). In these experiments, the crude EMV pellets and purified EMVs isolated by Optiprep^TM^ gradient were evaluated in parallel with corresponding bacterial cell pellets to begin to assess the degree of selectivity of partitioning of particular proteins into the EMVs. The extracellular GpbB (Banas and Vickerman, 2003) was readily identified in the crude vesicle pellet as well as in OptiPrep^TM^ gradient-purified EMVs. Only the WT strain’s EMVs were tested by Western blot with the anti-GbpB antibody because of its limited availability. The secreted negative regulator of biofilm cell density and genetic competence Smu_63c (Besingi et al., 2017) and the AtlA autolysin AtlA (Ahn and Burne, 2006) represented prominent components of both bacterial pellets and EMVs derived from all three strains. Interestingly, AtlA was not processed from its 107 kDa precursor form into its mature lower molecular weight 79 kDa form in the Δ*srtA* strain. While the integral membrane-localized chaperone insertases YidC1 and YidC2 (Palmer et al., 2012) were prominent in bacterial pellets, only trace antibody reactivity was occasionally observed against YidC1, and only in WT EMVs. Other co-translational protein transport machinery components, namely, the CM-associated signal recognition particle (SRP) pathway proteins Ffh and FtsY (Hasona et al., 2005), were sporadically visualized in bacterial pellets and purified MVs, but only of the WT, suggesting some potential carry-over of these proteins during vesicle formation in this strain. In contrast, SecA, the membrane-associated ATP-driven membrane-associated molecular motor protein of the post-translational general secretion pathway (GSP) (Lewis and Brady, 2015) was reproducibly observed in bacterial pellets of all three strains as well as in EMVs, particularly those of the WT and Δ*srtA* strains. This suggests that the GSP, more so than co-translational mechanisms, is involved in transport and loading of EMV cargo. Proteins with predicted secretion signals were more commonly found among EMV proteins, 7.79, 16.21, and 8.92%, for WT, Δ*srtA*, and Δ*sfp* strains, respectively, compared to 2.18, 2.41, and 2.96% of their corresponding CMs.

### *S. mutans* EMVs Have Fewer Lipoproteins and Integral Membrane Proteins With Fewer Transmembrane Domains Than Corresponding CMs

*Streptococcus mutans* membrane preparations are known to contain both integral membrane proteins that possess membrane-spanning segments, as well as CM-associated proteins that themselves lack TM domains (Mishra et al., 2019). Integral membrane proteins detected in EMVs and CMs were identified using the TMHMM web-based TM domain prediction tool^3^ (Krogh et al., 2001). The number of predicted TM domains for EMV and CM proteins of each strain is summarized (Table 2). This analysis revealed that CMs from all three strains contain numerous integral membrane proteins having from 1 to 16 TM domains. In stark contrast, far fewer integral membrane proteins were identified in the EMVs, and those detected contained only 1 or 2 TM domains with a sole exception, a 5 TM domain alkaline phosphatase (accession number AMF85662.1) observed only in the WT and Δ*sfp* strains (Supplementary Table S1). These bioinformatic results are consistent with the Western blot results described above in which the YidC1 and YidC2 components of the co-translational protein transport machinery, which act in concert with the *S. mutans* SRP pathway to insert larger multi-pass integral membranes (Mishra et al., 2019), were prevalent in whole bacterial samples, but absent or scant in corresponding EMVs. A paucity of trans-membrane spanning proteins has also been observed for *S. pyogenes* EMVs (Biagini et al., 2015). Our current results reinforce the growing recognition that bacterial CMs are not uniform (Faller, 2020), and strongly suggest that separate subsections of the *S. mutans* cell envelope are destined for vesicle secretion in contrast to other regions that support integral membrane protein insertion and CM biogenesis. It has been reported that *S. pyogenes* EMVs contained 28 lipoproteins, representing ∼72% of the total number of lipoproteins predicted by genome analysis of the strain studied (Biagini et al., 2015). We also explored the lipoprotein content of *S. mutans* EMVs. The *S. mutans* UA159 genome has been reported to encode 25 predicted lipoproteins (Bagos et al., 2008). Of these, 20, 21, and 19 were detected in CMs derived from the WT, Δ*srtA*, and Δ*sfp* strains, respectively, but only 10, 7, and 7 of the predicted lipoproteins were found in the corresponding EMVs. Thus, in contrast to *S. pyogenes* EMVs, lipoproteins represented only 4–10% of *S. mutans* vesicular proteome.

**TABLE 2:** Predicted number of transmembrane domains in proteins detected in EMVs compared to proteins detected in corresponding cytoplasmic membranes.

	WT				Δ*srtA*				Δ*sfp*			
	Membranes		Vesicles		Membranes		Vesicles		Membranes		Vesicles	
Predicted number of transmembrane domains	#	%	#	%	#	%	#	%	#	%	#	%
0	785	81.69	199	91.28	706	81.15	63	85.14	600	80.86	149	88.69
1	84	8.74	16	7.34	77	8.85	8	10.81	70	9.43	14	8.33
2	18	1.87	2	0.92	15	1.72	3	4.05	15	2.02	4	2.38
3	7	0.73	–	–	6	0.69	–	–	5	0.67	–	–
4	12	1.25	–	–	13	1.49	–	–	12	1.62	–	–
5	18	1.87	1	0.46	17	1.95	–	–	13	1.75	1	0.60
6	11	1.14	–	–	11	1.26	–	–	7	0.94	–	–
7	3	0.31	–	–	3	0.34	–	–	1	0.13	–	–
8	6	0.62	–	–	5	0.57	–	–	4	0.54	–	–
9	3	0.31	–	–	3	0.34	–	–	3	0.40	–	–
10	9	0.94	–	–	9	1.03	–	–	7	0.94	–	–
11	3	0.31	–	–	3	0.34	–	–	3	0.40	–	–
12	1	0.10	–	–	1	0.11	–	–	1	0.13	–	–
16	1	0.10	–	–	1	0.11	–	–	1	0.13	–	–

### Functional Analysis of *S. mutans* EMV and CM Proteins

**FIGURE 5:**
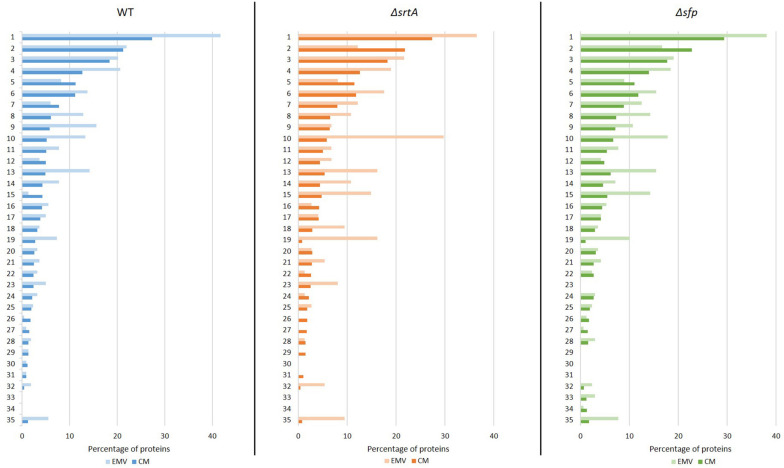
Functional annotation analysis of proteins detected in *S. mutans* EMVs compared to cytoplasmic membranes using Database for Annotation, Visualization, and Integrated Discovery (DAVID). Percentages of total *S. mutans* WT, Δ*srtA*, and Δ*sfp* EMV and total cytoplasmic membrane (CM) proteins in the top 35 functional categories are illustrated. (1) Metabolic pathways, (2) ATP binding, (3) transferase, (4) biosynthesis of secondary metabolites, (5) hydrolase, (6) metal-binding, (7) biosynthesis of amino acids, (8) RNA-binding, (9) ligase, (10) signal, (11) oxidoreductase, (12) kinase, (13) translation, (14) purine metabolism, (15) structural constituent of ribosome, (16) lyase, (17) pyrimidine metabolism, (18) isomerase, (19) glycolysis/gluconeogenesis, (20) protease, (21) GTP binding, (22) cell cycle/cell wall, (23) starch and sucrose metabolism, (24) NAD, (25) NADP, (26) flavoprotein, (27) helicase, (28) chaperone, (29) DNA replication, (30) glycerolipid metabolism, (31) stress response, (32) dental caries, (33) fatty acid metabolism, (34) lipid metabolism, and (35) pyruvate metabolism.

The proteins detected in EMV and corresponding CM samples were also subjected to functional analysis using the DAVID. A graphic representation of the 35 most highly represented functional categories is shown in Figure 5. The top category for both CMs and EMVs was metabolic pathways. This category contained a higher percentage of the total EMV proteins compared to the total CM proteins for each of the three strains. Other examples of functional categories that were over-represented in EMVs compared to CMs included biosynthesis of secondary metabolites (WT, Δ*srtA*, Δ*sfp*), RNA-binding (WT, Δ*srtA*, Δ*sfp*), ligase (WT, Δ*sfp*), signaling (WT, Δ*srtA*, Δ*sfp*), translation (WT, Δ*srtA*, Δ*sfp*), purine metabolism (WT, Δ*srtA*, Δ*sfp*), ribosomal proteins (Δ*srtA*, Δ*sfp*), isomerase (Δ*srtA*), glycolysis/gluconeogenesis (*WT*, Δ*srtA*, Δ*sfp*), starch and sucrose metabolism (*WT*, Δ*srtA*), dental caries (Δ*srtA*), and pyruvate metabolism (*WT*, Δ*srtA*, Δ*sfp*). In contrast, the ATP binding (Δ*srtA*, Δ*sfp*) and hydrolase categories (WT, Δ*srtA*, Δ*sfp*) were over-represented in CMs compared to corresponding EMVs. When relative levels of EMV proteins within each functional category were compared among the three strains, it is notable that deletion of *srtA* and *sfp* enhanced the percentage of proteins in the isomerase, signaling, ribosomal protein, glycolysis/gluconeogenesis, dental caries, and pyruvate metabolism categories. These shifts likely reflect physiological adaptations in the mutant strains as well as more direct impacts of SrtA and Sfp on EMV development.

### Lipidomic Comparison of *S. mutans* EMVs and CM Preparations

**FIGURE 6:**
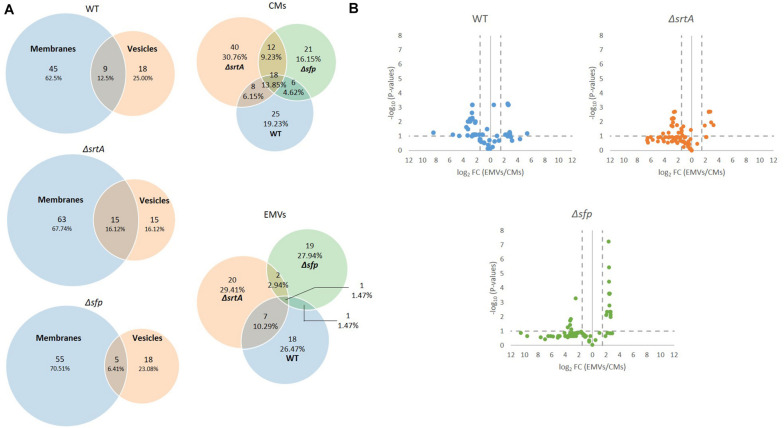
Comparison of EMV lipid content with corresponding cytoplasmic membranes from *S. mutans* wild-type and the mutant strains. **(A)** Venn diagram comparing lipids detected in cytoplasmic membranes (CM) and corresponding EMVs, and in all three strains’ EMVs and CMs (right). **(B)** Volcano plots of *p*-values vs. fold-change to compare lipid profiles of CMs (left of *Y*-axis) with corresponding EMVs (right of *Y*-axis) from each strain.

Quadruplicate EMV and CM samples from each of the three strains were also subjected to lipidomic analysis by high-resolution LC-MS on a Bruker Impact II Q-TOF MS system. This experiment identified both lipids and metabolites, with data further analyzed and curated using the MetaboAnalyst 4.0 web tool (Chong et al., 2019) to identify features and significant changes between WT and mutants. A given lipid was considered as present if it was detected in three of four replicates. Lipids were quantitated from the LC-MS intensity profiles of the intact lipids. No MS/MS spectra were acquired; therefore, all lipid identifications are matched by accurate mass measurement and only the general lipid identification is reported (i.e., PC 40:3, with no distinguishing the specific carbon chain lengths on the fatty acyl/alkyl chains). Future experiments will include LC-MS/MS to elucidate specific lipid identities; however, for the purpose of this discovery-based lipidomic analysis, LC-MS on a high-resolution, high-mass accuracy instrument has provided newly discovered lipid classes associated with *S. mutans* vesiculogenesis. A total of 130 individual lipids were collectively identified in CMs from the three strains, and 68 in total in the EMVs (Supplementary Table S2). The degree of overlap in lipid composition of EMVs compared to CMs for each strain, as well as three-way comparisons of EMVs and CMs from all three strains, are illustrated by Venn diagram (Figure 6A). More total lipids were identified in the Δ*srtA* strain (*n* = 78) compared to the WT (*n* = 54) and Δ*sfp* (*n* = 60) strains in CM. The number of different lipids identified in WT, Δ*srtA*, and Δ*sfp* EMVs was 27, 30, and 23, respectively. When the stringency for consideration of a given lipid’s presence was reduced to 2/4 or 1/4 replicates, the total number of different lipids identified in the CMs was increased to 64, 162, and 109, or 82, 184, and 142, and in the EMVs was increased to 34, 56, and 43, or 48, 87, 59 for the WT, Δ*srtA*, and Δ*sfp* strains, respectively. Thus, lowering the stringency for inclusion in the dataset appears to increase the propensity to identify more lipids at various stages along biosynthetic pathways, which is more obvious in the Δ*srtA* mutant. Higher percentages of lipids were exclusive to CMs, ∼63, 68, and 71% for the WT, Δ*srtA*, and Δ*sfp* strains, respectively, compared to the percentages of lipids exclusive to their EMVs, ∼24, 16, and 23% (Figure 6A). Approximately 13, 16, and 6% of lipids were shared between CMs and EMVs for the WT, Δ*srtA*, and Δ*sfp* strains, respectively. Of note, substantially higher percentages of lipids were identified as exclusive to EMVs compared to the percentages of proteins exclusive to the EMVs from each strain (compare Figures 3A, 6A). Also, in contrast to the proteomic data, there was notably less overlap in lipid composition among CMs from the three strains (14%) than there was in protein composition (72%). This was also true of three-way overlap in EMV lipid composition (1%) compared to three-way overlap in protein composition (28%). Taken together these data reveal that not only is the EMV lipid content more dissimilar among the three strains tested than is CM lipid content, the CM and EMV lipid content is more dissimilar for each strain than is their protein content.

### Lipid Categories and Classes Vary Between Strains and Between EMVs and CMs

**FIGURE 7:**
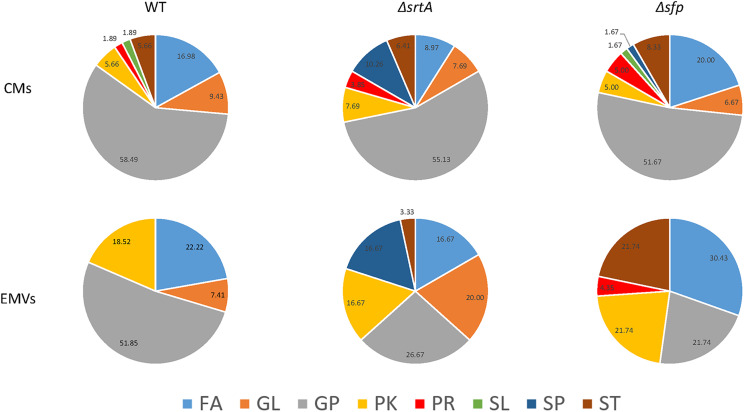
Distribution of lipids detected in cytoplasmic membranes as compared to EMVs of *S. mutans* wild-type and the mutant strains. FA, fatty acyls; GL, glyceroplipids; GP, glycerophospholipids; PK, polyketides; PR, prenol lipids; SL, saccharolipids; SP, sphingolipids; ST, sterol lipids. Percentages of lipids in each category are indicated in the pie charts.

Lipids are classified into eight categories, fatty acyls, glycerolipids, glycerophospholipids, sphingolipids, sterol lipids, prenol lipids, saccharolipids, and polyketides, of which each contain distinct classes and subclasses (Fahy et al., 2005). This system is chemically based and takes into consideration distinct hydrophobic and hydrophilic elements, with each unique lipid given a 12-digit identifier. The relative distribution of lipids from each of these categories was determined for CMs and corresponding EMVs for each strain (Figure 7A). The majority of lipids identified in CMs from each strain were glycerophospholipids. In contrast, only EMVs from the WT strain contained a preponderance of glycerophospholipids, which were notably decreased in EMVs from the Δ*srtA* and Δ*sfp* mutants. Fatty acyls were a prominent lipid category in all EMVs, especially those of the Δ*sfp* strain, but were diminished in Δ*srtA* CMs compared to the other two strains. EMVs of all three strains were notably enriched in polyketides compared to the corresponding CMs. While not found in EMVs or CMs of the WT, sphingolipids were readily observed in both EMVs and CMs of the Δ*srtA* mutant, and detected in low amounts in CMs of the Δ*sfp* strain. Sterol lipids were detectable at similar levels in CMs of all three strains, but this class was not found in WT EMVs and was prominent in Δ*sfp* EMVs. Δ*sfp* was also the only strain to contain prenol lipids in its EMVs. Lacking, however, from Δ*sfp* EMVs were glycerolipids, which were increased in Δ*srtA* EMVs compared to Δ*srtA* CMs. Sterol lipids were detected in low amounts in WT and Δ*sfp* CMs, but not in any strain’s EMVs or in Δ*srtA* CMs. Overall the proportions of glycerophospholipids, polyketides, fatty acyls, and glycerolipids appeared similar between WT CMs and EMVs, whereas prenol lipids, saccharolipids, and sterol lipids were lacking from WT EMVs. In contrast to the WT, the proportion of each lipid category was more obviously divergent between Δ*srtA* and Δ*sfp* EMVs compared to their corresponding CMs. These data indicate that the relative proportion of each lipid category varies between CMs and EMVs, and that this variation is markedly increased when genes encoding SrtA and Sfp are deleted. This may stem in part from cellular adaptation to the physiological disruption of losing these protein’s enzymatic functions, or alternatively from more direct impacts they may contribute to lipid synthesis pathways.

**FIGURE 8:**
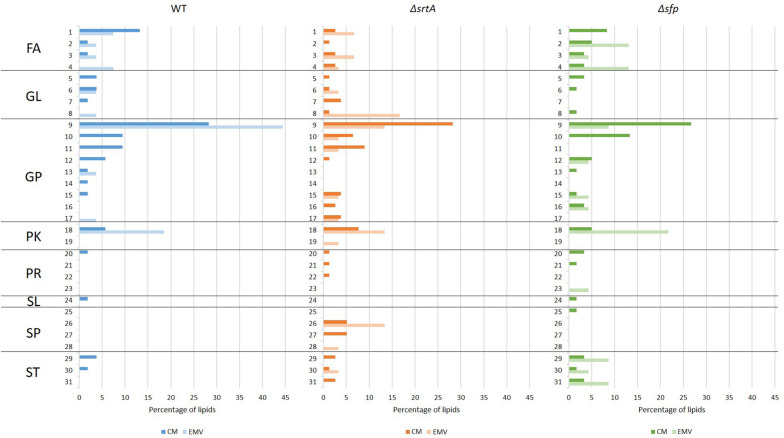
Categories and main classes of lipids detected in cytoplasmic membranes (CMs) compared to EMVs of *S. mutans* wild-type, Δ*srtA*, and Δ*sfp* strains. (1) FA, fatty acids and conjugates; (2) FA, eicosanoids; (3) FA, fatty esters; (4) FA, fatty amides; (5) GL, DAG diacylglycerol; (6) GL, MAG monoacylglyceride; (7) GL, glycosyldiradylglycerols; (8)GL, TAG triacylglycerol; (9) GP, cardiolipin; (10) GP; PI phosphatidylinositol; (11) GP, PS phosphatidylserine; (12) GP, PG glycerophosphoglycerols; (13) GP, glycerophosphoinositolglycans; (14) GP, glycosylglycerophospholipids; (15) GP, PA phosphatidic acid; (16) GP, PC phosphatidylcholine; (17) GP, PE phosphatidylethanolamine; (18) PK, flavonoids; (19) PK, non-ribosomal peptide/polyketide hybrids; (20) PR, isoprenoids; (21) PR, hopanoids; (22) PR, polyprenols; (23) PR, quinones and hydroquinones; (24) SL, acyltrehaloses; (25) SP, ceramides; (26) SP, neutral glycosphingolipids; (27) SP, phosphosphingolipids; (28) SP, sphingoid bases; (29) ST, sterols; (30) ST; bile acids and derivatives; (31) ST, secosteroids. FA, fatty acyls; GL, glyceroplipids; GP, glycerophospholipids; PK, polyketides; PR, prenol lipids; SL, saccharolipids; SP, sphingolipids; ST, sterol lipids.

Lipids were also evaluated according to main class (Figure 8). This analysis revealed that cardiolipin was the most prevalent class of glycerophospholipids in WT, Δ*srtA*, and Δ*sfp* CMs, as well as in WT EMVs. Eicosanoids, fatty amides, and particularly flavonoids were more prevalent than cardiolipins in Δ*sfp* EMVs. In contrast to the WT strain, cardiolipins were more prevalent in Δ*srtA*’s CMs rather than in this strain’s EMVs. Similar to the Δ*sfp* strain, flavanoids were also prevalent in WT and Δ*srtA* EMVs compared to their CMs. Neutral sphingolipids were only identified in the Δ*srtA* strain, and were more prevalent in EMVs compared to CMs. Triacylglycerides were also present at a relatively high level in Δ*srtA* EMVs (∼17%), but were absent or scant (<4%) in the other strain’s EMVs or in any strain’s CMs. Fatty acids and conjugates were more prevalent in WT and Δ*sfp* CMs than those of the Δ*srtA* strain, and more prevalent in Δ*srtA* EMVs compared to WT EMVs. This lipid class was absent from Δ*sfp* EMVs. Steroids and secosteroids were detected in EMVs of the Δ*sfp* mutant, but not in EMVs of the other two strains, and were more abundant in Δ*sfp* EMVs (∼9%) than in Δ*sfp* CMs (∼3%). Collectively, these data reiterate the results observed at the lipid category level, and identify the lipid classes that differ between strains and between CMs and EMVs, again highlighting that such variation is more apparent in the Δ*srtA* and Δ*sfp* mutants.

Volcano plots of fold-change vs. *p*-value also revealed differences in lipid composition between CMs and EMVs of each strain (Figure 6B). This analysis illustrates that there was a greater number of over-represented lipids in the Δ*sfp* EMVs compared to the WT and the Δ*srtA* strains. Individual lipids exclusive to or over-represented in EMVs (log2 > 1.5) of each strain are listed in Supplementary Table S3. WT EMVs contained 12 over-represented lipids including three cardiolipins, five flavonoids, two fatty acyls, one glycerophosphoinositolglycan, and one glycerolipid. Fewer individual lipids were over-represented in the Δ*srtA* EMVs compared to the WT and Δ*sfp* strains. The Δ*srtA* EMVs contained six over-represented lipids in five different categories including one phosphotidylinositol, one flavonoid, two fatty acyls, one triacylglycerol, and one sphingolipid. At *n* = 13, the Δ*sfp* strain had the largest number of over-represented lipids, five polyketides (all flavonoids), three sterol lipids, one glycerophospholipids, three fatty acyls, and one prenol lipid.

Lipid architecture plays an important role in environmental adaptation (Fozo and Quivey, 2004). The cariogenic pathogen *S. mutans* is highly tolerant of the acidic environment it produces during fermentation of dietary carbohydrates to organic acid end products (Fozo et al., 2007). This acid tolerance is facilitated by a membrane composition rich in monounsaturated long-chain fatty acids (Fozo and Quivey, 2004). Our results revealed that both the *S. mutans* CM and EMV lipidome is composed exclusively of long-chain fatty acids, with as many as 80 carbons in the carbon chains (Table 3). Interestingly, varying degrees of unsaturation were observed in CM compared to EMV samples. Polyunsaturated lipids predominated in CMs from all strains, compared to monounsaturated (one double bond) and saturated fatty acyl chain (zero double bonds) lipids. The number of individual lipid double bonds reached up to 11 for WT, 14 for Δ*srtA* strains, and 17 for the Δ*sfp* mutant (Supplementary Table S2). The Δ*srtA* CM sample was highest in monounsaturated lipids (26.32%) compared to the WT and Δ*sfp* strains (17.14 and 16.22%, respectively). The distribution of saturated lipids within the EMVs of the three strains varied considerably. In WT EMVs, polyunsaturated lipids represented 55.56% of lipids having fatty acyl chains, with 16.67% being monounsaturated, and 27.78% saturated. However, in Δ*srtA* EMVs, the level of saturation was more evenly distributed, with 31.58% each of poly- and monounsaturated lipids, and 36.84% of saturated lipids. In striking contrast, Δ*sfp* EMVs contained exclusively polyunsaturated lipids.

**TABLE 3:** Fatty acyl and saturation profiles of lipids contained in *S. mutans* EMVs and corresponding cytoplasmic membranes.

EMV								
Strain	Total lipids in sample	Lipids with fatty acyl chains	Saturated		Monounsaturated		Polyunsaturated	
	#	#	#	%	#	%	#	%
WT	27	18	5	27.78	3	16.67	10	55.56
Δ*srtA*	30	19	7	36.84	6	31.58	6	31.58
Δ*sfp*	23	5	0	0.00	0	0.00	5	100.00

**CM**								

**Strain**	**Total lipids in sample**	**Lipids with fatty acyl chains**	**Saturated**		**Monounsaturated**		**Polyunsaturated**	
	**#**	**#**	**#**	**%**	**#**	**%**	**#**	**%**

WT	54	35	10	28.57	6	17.14	19	54.29
Δ*srtA*	78	57	15	26.32	15	26.32	27	47.37
Δ*sfp*	60	37	12	32.43	6	16.22	19	51.35

## Discussion

The goals of this study were to characterize biophysical properties and the protein and lipid composition of EMVs from *S. mutans* and to examine the roles of SrtA and Sfp in vesiculogenesis and impact on *S. mutans* EMV properties and composition. The results showed that *S. mutans* EMV cargo is enriched in proteins known to contribute to biofilm formation and cell envelope architecture. Bacteriocin Smu_1904c was found in EMVs, but not CMs, of the WT and its *srtA* and *sfp* mutant strains. This result is consistent with findings in *Lactobacillus acidophilus* in which membrane vesicles were identified as a delivery vehicle for bacteriocin (Dean et al., 2020). Also prevalent in *S. mutans* EMVs were glucosyl hydrolases, Gtfs, and Gbps, all of which are well established *S. mutans* virulence attributors that play a significant role in facilitating biofilm formation (Lemos et al., 2019). GTFs were recently reported to be carried by *S. mutans* EMVs, and impaired biofilm formation by a *gtfBC* mutant was restored by exogenous addition of EMVs harvested from broth culture supernatant of the parent strain (Senpuku et al., 2019). Addition of *S. mutans* EMVs also upregulated biofilm formation by early tooth colonizers including *Streptococcus mitis*, *Streptococcus oralis*, *Streptococcus sanguinis*, *Streptococcus gordonii*, *Actinomyces naeslundii*, and *Actinomyces oris*, while biofilm formation by other microbial species including *Candida albicans*, *S. salivarius*, *S. aureus*, *Streptococcus anginosus*, *Streptococcus intermedius*, and *S. pyogenes* were not affected (Senpuku et al., 2019). Hence, *S. mutans* EMVs appear to support biofilm formation not only by *S. mutans* itself, but also by others that may reside in close proximity in the oral cavity.

In the current study, another consistently identified protein in each strain’s EMVs was the AtlA autolysin (Ahn and Burne, 2006). AtlA’s prevalence suggests that its peptidoglycan-degrading activity contributes to *S. mutans* vesiculogenesis. In *S. pyogenes*, EMV formation is enhanced by incorporation of a sublethal concentration of the peptidoglycan synthesis inhibiting antibiotic penicillin in the growth medium (Biagini et al., 2015). The peptidoglycan hydrolase SagA/GbpB was also identified in EMVs of the WT *S. mutans* strain. For unknown reasons, AtlA was not processed to its active lower molecular weight form in the Δ*srtA* strain. Because AtlA plays a critical role connecting cell surface biogenesis, biofilm formation, genetic competence, and autolysis (Ahn and Burne, 2006), this finding helps to explain the significantly impaired *S. mutans* biofilm formation previously observed when *srtA* is deleted (Oli et al., 2012). EMVs from the Δ*srtA* strain contained the fewest number of uniquely detected proteins, while those of the Δ*sfp* mutant contained the highest number. Among the proteins found only in Δ*sfp* EMVs were BacA and BacA2 that, like Sfp, are also encoded in the *S. mutans* TnSmu2 genomic island (Knox et al., 1986). BacA shares some homology with surfactin synthetase, although *S. mutans* is not known to produce surfactin. BacA2 is a NRPS that functions, and is annotated as, a bacitracin synthetase in other organisms. BacA and BacA2 were detected in CMs, but not EMVs, of the WT strain. BacA was present but not over-represented in Δ*srtA* EMVs. This suggests that Sfp may contribute to regulation of expression or localization of other gene products encoded within this locus. Also uniquely detected in Δ*sfp* EMVs was BrpA. BrpA is a surface associated protein with important roles in cell envelope biogenesis, biofilm formation and virulence (Wen et al., 2006, 2018). Deficiency of this protein in *S. mutans* impairs biofilm formation (Wen et al., 2006), affects cell division and membrane fatty acid composition thereby reducing acid tolerance, and significantly diminishing the organism’s ability to compete within polymicrobial communities and to cause dental caries in a rat model (Wen et al., 2018).

We found the average sizes of *S. mutans* EMVs to be in the typical range of those reported for other bacteria, including Gram-positive organisms (Brown et al., 2015). We also observed a bimodal distribution of small and large vesicles for all the *S. mutans* strains tested, similar to that observed for *B. subtilis* (Brown et al., 2014) and *S. pyogenes* (Biagini et al., 2015). This may be due to fusion or agglomeration of EMVs. ζ-Potential measurements of isolated EMVs from all three *S. mutans* strains approached electroneutrality, in contrast to corresponding protoplasts that were more negatively charged. Thus, electrostatic repulsion would support a uniform colloidal suspension of the protoplast preparations, but not the EMV samples. Irrespective of the method used for analysis, the Δ*srtA* EMVs were consistently larger than those of the other two *S. mutans* strains analyzed. This may stem from the alteration in AtlA processing observed in Δ*srtA* mutant, and/or from the altered lipid composition of this mutant compared to the other two strains. For example, as shown in Figure 8, neutral sphingolipids and triacylglycerides were prominent in Δ*srtA* EMVs but not in those of other two strains. Due to its unusual structural properties, cardiolipin can influence membrane curvature (Schlame, 2008), and sterol lipids are known to confer membrane rigidity and stiffness (Faller, 2020). Thus, lipid composition of EMVs from any given strain would be expected to potentially impact their size and biophysical properties.

We did not observe the hyper-vesicle phenotype reported for Δ*sfp* strains of *B. subtilis* (Brown et al., 2014). While Sfp contributes to surfactin (subtilisin) biosynthesis in *B. subtilis*, there is no evidence that *S. mutans* produces surfactin, although it is susceptible to that produced by other oral streptococci (Kreth et al., 2005; Okahashi et al., 2013). In contrast to *B. subtilis*, the *S. mutans*Δ*sfp* sample yielded the least EMV particles among the three strains tested, although total protein assay measurements appeared higher for the Δ*sfp* EMV preparations than those of the other strains (not shown). Flavonoids are known to cause misleadingly high Bradford protein assay readings (Compton and Jones, 1985), and we found WT, Δ*srtA*, and particularly Δ*sfp* EMVs to be enriched in flavonoids.

Considerably more overlap was observed among the EMV and CM proteomes of the three *S. mutans* strains tested, than among their corresponding lipidomes. Lipidomic analysis revealed that, similar to EMVs of *S. pyogenes* (Resch et al., 2016), WT *S. mutans* EMVs were enriched in cardiolipin compared to CMs, but the opposite was true of EMVs from the Δ*srtA* and Δ*sfp* strains (see Figure 8). Sphingolipids were only detected in membranes of the Δ*srtA* strain and were more prevalent in EMVs than in the CM, while sterol lipids were over-represented in EMVs from the Δ*sfp* strain, and glycerolipids were absent from Δ*sfp* EMVs altogether (see Figure 7). In contrast to *S. pyogenes* (Biagini et al., 2015) and *S. pneumoniae* (Olaya-Abril et al., 2014), *S. mutans* EMVs were not enriched in lipoproteins. Also striking was the limited number of TM domains within EMV membrane proteins compared to those detected in corresponding CM preparations (see Table 2). This finding is consistent with the relative distribution of components of the co-translational membrane protein insertion machinery including YidC1, YidC2, FFh, and FtsY (Mishra et al., 2019), which were prominent in whole cell extracts but scant or lacking in EMVs. In contrast, SecA, the ATP-dependent molecular motor component of the GSP (Lewis and Brady, 2015) was readily detected in EMVs of the WT and Δ*srtA* strains, as well as in cellular extracts of all three strains. Extracellular secreted proteins, such as those detected in *S. mutans* EMVs would be expected to be transported by the GSP. Our results demonstrate a partitioning of specific protein transport pathways during *S. mutans* EMV compared to CM biogenesis. In *S. pyogenes*, a specialized microdomain called the ExPortal is reported to contribute to the secretion and maturation of extracellular proteins (Rosch and Caparon, 2005). This region is rich in anionic lipids (Rosch et al., 2007), and has also been associated with peptidoglycan synthesis (Vega et al., 2013). *S. pyogenes* EMVs have been postulated to derive from the ExPortal (Biagini et al., 2015). In *S. mutans*, SecA and Sortase also co-localize to a discrete microdomain similar to the *S. pyogenes* ExPortal (Hu et al., 2008), and it has been reported that *S. mutans* virulence factors such as P1 (aka SpaP, PAc, AgI/II), glycosyltransferase, and fructosyltransferase utilize this microdomain for secretion particularly during growth in biofilms (Huang et al., 2008). Bacterial co-translational and post-translational protein transport pathways converge at the membrane-localized SecYEG translocon (Ryabichko et al., 2020). While SecA is generally associated with post-translational protein transport, it can also associate with ribosome nascent chain complexes to support co-translational transport (Huber et al., 2017). SecA has also been reported to oligomerize and interact with a dimeric SecYEG complex (Gold et al., 2010). The stability of SecYEG dimers and SecA oligomers is severely compromised following the engineered absence of cardiolipin in the inner membrane of *Escherichia coli*, thereby impeding translocation and insertion of several known protein substrates (Ryabichko et al., 2020). Thus, the relative proportions of cardiolipin in the *S. mutans* EMVs compared to corresponding CMs (higher in WT EMVs, lower in Δ*srtA* and Δ*sfp* EMVs) would impact the efficiency of secretion, or membrane protein insertion, of substrates that depend on dimerization of SecYEG and/or oligomerization of SecA. This in turn could drive segregation of particular proteins into EMVs destined for secretion, or into membrane regions destined to remain within the CM. Bacterial membrane microdomains have been reported to concentrate proteins involved in secretion, signal transduction, and metabolism regulation (Lopez and Koch, 2017; Nagakubo et al., 2020), and have been previously mentioned as a possible gate for vesicular secretion (Biagini et al., 2015). Lipid rafts are more ordered sections of membrane and are enriched in integral membrane proteins and sterol lipids that would increase membrane stiffness and thickness (Faller, 2020). Except for the Δ*sfp* strain whose EMVs were enriched for sterol lipids, this class was absent or less prevalent in EMVs compared to corresponding CMs in the other two strains. Thus, the lipid composition of membrane microdomains that supports *S. mutans* veisculogenesis likely confers greater membrane fluidity.

Polyketides, predominantly flavonoids, were consistently over-represented in EMVs compared to corresponding CMs of all three *S. mutans* strains tested. Several hundreds of *S. mutans* strains have now been sequenced, and most are considered to harbor the TnSmu2 genomic island or other NRP synthetase/PK synthase gene clusters involved in oxygen and H_2_O_2_ tolerance (Wu et al., 2010). The reported end product of these loci, a hybrid NRP/PK pigment identified by HPLC separation of methanol extracts of whole bacterial cells, was detected in EMVs of the Δ*sfp* mutant, but not in samples from the WT or Δ*srtA* strains. Genomic islands such as TnSmu2 are proposed to confer a selective advantage to the bearer organisms under particular environmental conditions, including the presence of competing organisms. The human oral cavity is especially hostile and includes numerous organisms capable of killing *S. mutans* via surfactin (Kreth et al., 2005; Okahashi et al., 2013) or H_2_O_2_ (Loo et al., 2000) production. Thus, contents of EMVs could enable *S. mutans* to overcome the toxic products of other local bacteria, as well as to directly inhibit competing organisms by carriage of antimicrobial bacteriocins (mutacins) and flavonoids. Flavonoids produced by plants have long been recognized for their antimicrobial properties (Panche et al., 2016). While bacteria have been used as vehicles for flavonoid production (Zha et al., 2019), whether any species possess *de novo* synthesis capacity has not to our knowledge been established. It is unclear if the presence of flavonoids in our samples represents endogenous synthesis or reflects products of metabolic degradation.

Our lipidomic analyses showed that *S. mutans* EMVs, as well as their corresponding CMs, contain exclusively long chain and very long chain fatty acids, the majority of which are unsaturated. These findings are in contrast to lipidomic studies of *S. pneumoniae* in which EMVs contained short-chain lipids (Olaya-Abril et al., 2014). It has been shown that *S. mutans* needs long chain fatty acids (Fozo and Quivey, 2004) as well as unsaturated fatty acids (Bojanich and Calderón, 2017) to survive in an acidic environment. Glycerophospholipids were the predominant lipid class in all samples except for Δ*sfp* EMVs, in which the predominant classes were fatty acyls, followed by glycerophospholipids and sterols (see Figure 7). As stated above, the most abundant lipid in WT EMVs and CMs was cardiolipin, a glycerophospholipid derived from phosphatidylglycerol (PG) (De Vrije et al., 1988), and synthesized by cardiolipin synthase. The amount of cardiolipin in Δ*srtA* and Δ*sfp* EMVs was notably lower than in WT EMVs, even though cardiolipin synthase was detected in CMs of all three strains. In contrast to the monounsaturated lipid profile reported for *S. mutans* during chemostat growth under acidic conditions (Fozo and Quivey, 2004), our results showed a preponderance of monounsaturated and polyunsaturated lipids in both CMs and EMVs. This disparity may stem from differences in growth conditions or lipid detection methodologies, but suggests that the presence of unsaturated lipids in *S. mutans* membranes is more common than previously recognized.

Several studies have now described the utility of EMVs produced by *B. anthracis* (Rivera et al., 2010), *Neisseria meningitidis* (Schwechheimer and Kuehn, 2015), *S. aureus* (Wang et al., 2018), *S. pneumoniae* (Brown et al., 2015), and *S. mutans* (Senpuku et al., 2019) as potential delivery vehicles for vaccine antigens. Intranasal immunization of Balb/c mice with *S. mutans* EMVs in concert with the TLR3 agonist poly(I-C) was an effective method to raise anti-GtfC IgA and IgG antibodies (Nakamura et al., 2020). Thus, comprehensive information regarding the content and characteristics of *S. mutans* EMVs will be useful in future studies to evaluate their role in induction of protective immunity, and to facilitate studies regarding their specific contributions to mono and mixed species biofilms development.

Taken together our current results add to the understanding of EMV biogenesis in *S. mutans*. Both SrtA and Sfp influence the protein, and especially the lipid content, of both EMVs and CMs. Protein transport pathway components are differentially localized in EMVs compared to CMs and this partitioning appears to be influenced by lipid, including cardiolipin, distribution. While *S. mutans* shares features with EMVs of other Gram-positive bacteria, unique features were identified as well including a high proportion of long chain fatty acids and a lower proportion of lipoproteins than EMVs produced by other streptococcal species. This new information extends our understanding of the biology of this remarkably resilient and tenacious oral biofilm dweller.

## Data Availability Statement

The proteomic and metabolomic data have been submitted to the (project PXD019825) and MetaboLights repository respectively (MTBLS1812).

## Author Contributions

JM-A, KB, ZW, and LJB conceived experiments. JM-A, AB-B, KB, and LJB designed experiments and research methodology. JM-A, PL, AB-B, and MK performed the research and data collection. JM-A, SM, KB, and LJB analyzed the data. JM-A and LJB wrote the manuscript. All authors reviewed the manuscript drafts and inputted corrections, amendments, and their expertise.

## Conflict of Interest

The authors declare that the research was conducted in the absence of any commercial or financial relationships that could be construed as a potential conflict of interest.

## Footnotes

1 https://www.ncbi.nlm.nih.gov/genome/?term=txid210007[Organism:noexp]

2 https://www.metaboanalyst.ca/home.xhtml

3 http://www.cbs.dtu.dk/services/TMHMM/
